# Daily Affect and Self-Esteem in Early Adolescence: Correlates of Mean Levels and Within-Person Variability

**DOI:** 10.5334/pb.467

**Published:** 2019-02-18

**Authors:** Sabine Nelis, William M. Bukowski

**Affiliations:** 1 KU Leuven, Leuven, BE; 2 Concordia University, Montreal, CA

**Keywords:** daily affect, self-esteem, early adolescence, internalizing symptoms, peers, aggression

## Abstract

Emotions and self-esteem are critical components of well-being and adaptation during adolescence. People differ in their average levels of affect and self-esteem, as well as in how much their affect and self-esteem fluctuate from moment to moment. Fluctuations in affect in particular have not been extensively examined in relation to adolescent-relevant variables. The present study investigates internalizing symptoms, social functioning, and overt and relational aggression as correlates of average levels and within-person variability in daily positive and negative affect (PA and NA) and self-esteem. Crucially, unique association were examined controlling for the other variables. Early adolescents (mean age 10.8 years, *N* = 94) completed daily diaries across four days on PA, NA, and self-esteem. They also completed general questionnaires, as did peers. Some key findings were that more internalizing symptoms were significantly associated with more variability in NA. The importance of peer relationships for adolescents’ daily mean levels of PA and NA were shown. Peer-perceived social functioning was associated with less fluctuations in self-esteem. Some unexpected, non-significant, findings for aggression appeared. Finally, higher mean NA were associated with more NA fluctuations, whereas higher mean PA and self-esteem were associated with less fluctuations.

Emotions and self-esteem are critical components of well-being and adaptation during adolescence. A crucial research aim concerns the identification of factors that potentially influence adolescent’s affective experiences and self-esteem. The basic premise of the study is that individual differences in *mean levels* represent only one aspect of affective experiences and self-esteem. Individuals also differ in the degree to which they *fluctuate* in their affect and self-esteem. The validity of this distinction has been shown in studies with adults (Franck et al., 2016; Houben, Van Den Noortgate, & Kuppens, 2015) but has not been extensively examined in early adolescence. The purpose of the present study was to assess how mean levels as well as within-person variability of affect and self-esteem are associated with internalizing symptoms, social functioning, and aggression in early adolescence. A multi-informant method that relied on assessments from adolescents and their peers was used to examine theory-based hypotheses about internalizing problems, social functioning, aggression, and within-person variation in affect and self-esteem.

## Positive and Negative Affect

Positive affect (PA) refers to the experience of pleasant emotional states such as happiness and enthusiasm; whereas negative affect (NA) refers to the experience of unpleasant states such as sadness and anger (Watson & Clark, 1994). Positive and negative emotional experiences both have their unique neural activation pattern, besides a common cerebral network (Habel, Klein, Kellerman, Shah, & Schneider, 2005) and are, accordingly, known to have their own specific functions and benefits. For instance, PA, compared to NA, stimulates one’s momentary repertoire of thoughts and actions and creative thinking (Fredrickson, 1998) and promotes physical health (e.g., Moskowitz, 2003).

Researchers have argued that for a full understanding of emotions, state levels of emotions should be recognized and assessed in daily life across multiple time points (e.g., Houben et al., 2015). This ecologically valid approach allows for a rich understanding of positive and negative emotional experiences across multiple time points because it captures fluctuations in emotions over time. People with the same mean level of affect can substantially differ in how much their affect fluctuates around that mean. As indicated by Kuppens, Oravecz, and Tuerlinckx (2010), the fact that affect can change over time is a key aspect of affect (regulation). Strong variability in affect can be an indication of extreme affective reactivity to situational or internal triggers. One could argue that measures of within-person variation in affect and self-esteem are the most direct indicators of regulation. Thus, to more fully understand our emotional system, within-person variations also need to be assessed (Houben et al., 2015; Wichers, Wigman, & Myin-Germeys, 2015). Indeed, fluctuations in affect are associated with negative psychological outcomes over and above the role of the mean level of affect (Gruber, Kogan, Quoidbach, & Mauss, 2013; Neumann, van Lier, Frijns, Meeus, & Koot, 2011). Research on within-person variations in affect has increased in the last decades and it is known that early adolescents can differentiate between the experience of PA and NA (e.g., 8 to 11 years in Leonhardt, Könen, Dirk, & Schmiedek, 2016). However, research on within-person variations in affect in children and early adolescents remains scarce but is key. That is, emotional problems start to increase, and emotional regulation strategies are in development and continue to mature in adolescence (Ahmed, Bittencourt-Hewitt, & Sebastian, 2015).

## Self-Esteem

Self-esteem also forms an essential aspect of an individual’s well-being (Bukowski & Raufelder, 2018; Kernis, 2006). Self-esteem refers to an individual’s general level of self-worth or global evaluation concerning how much he or she likes him- or herself. This could be interpreted in terms of being a good person, being confident of oneself, and/or feeling happy with the way one is (Bukowski & Raufelder, 2018; Harter, 1982; Rosenberg, 1986). Although people do not feel equally skilled across all domains (e.g., social, academic, physical), self-esteem functions as a superordinate construct that is determined by one’s perceptions of functioning in more specific areas.

Comparable to emotions, a person’s level of self-esteem can vary across time with some individuals showing more fluctuations than others (Kernis, Cornell, Sun, Berry, & Harlow, 1993). Self-esteem stability refers to “the magnitude of short-term fluctuations that people experience in their contextually based, immediate feelings of self-worth” (Kernis, 2005, p. 1572). Both mean levels and higher fluctuations in self-esteem have been associated with unhealthy behaviors and poorer adjustment in several domains, for instance binge eating (Sanftner & Crowther, 1998). Adolescents face the developmental task of exploring their identities. Self-esteem increases during adolescence (Erol & Orth, 2011; Greene & Way, 2005) and becomes more stable with increased age, indicating that young ages might be especially salient for an examination of the fluctuations in self-esteem (Meier, Orth, Denissen, & Kühnel, 2011).

## Correlates of Daily Positive Affect, Negative Affect, and Self-Esteem

***Internalizing symptoms.*** Internalizing symptoms refer to the symptoms of anxiety and mood disorders. Depressive symptoms and generalized anxiety disorder tend to increase throughout adolescence (Copeland, Angold, Shanahan, & Costello, 2014; Ge, Conger, & Elder, 2001). Lower mean values of daily PA and higher mean daily NA (referring to an aggregation of affect measures across multiple time points) have been related to more depressive and anxiety symptoms, and depression in children and adolescents (Forbes, Williamson, Ryan, & Dahl, 2004; Larson, Raffaeilli, Richards, Ham, & Jewell, 1990; Neumann et al., 2011; Silk, Steinberg, & Morris, 2003; Van Roekel et al., 2016) as well as in adults (e.g., Thompson et al., 2012).

Whereas increased negative affect and decreased positive affect are diagnostic symptoms of depression, daily affective experiences also receive research interest to examine non-static aspects of affect in relation to psychopathology (Houben et al., 2015) as well as to examine the involvement of static and dynamic affective experiences in the *etiology* of psychopathology (Neumann et al., 2011). Interestingly, depressive symptoms have been associated with increased within-person variability in both PA and NA in adolescence; with within-person variability operationalized as the standard deviation of repeated affect measures (Larson et al., 1990; Silk et al., 2003; Van Roekel et al., 2016). Moreover, variability in both PA and NA have been related to prospective anxiety symptoms (Neumann et al., 2011). Although this indicates that too much emotional variability is not adaptive in adolescence, research in a group of early adolescents at the end of primary school remains scarce and requires replication.

Concerning self-esteem, it is well-known that having a low general self-esteem is closely linked to depression and anxiety (Sowislo & Orth, 2013). Nonetheless, it has been shown, majorly in students and adults, that within-person variations in self-esteem are even more strongly linked to depressive symptoms than the average level of self-esteem (Butler, Hokanson, & Flynn, 1994; Franck & De Raedt, 2007; Franck et al., 2016). Self-esteem variability may put individuals more at risk for depression as it represents a stronger reactivity to daily (stressful) experiences. Fluctuations in self-esteem have been seen as a strong response to events, either external or self-generated, that are evaluative and interpreted as being relevant for the individual’s self-esteem (Kernis, 2005). As such, a stronger response to events that potentially threaten self-esteem or positive self-views is assumed to be expressed by increased fluctuations in self-esteem.

***Social context: Social functioning and peer relationships.*** Peer relationships play a crucial role in development and are protective against risk for several problems, including victimization, non-optimal family experiences, and internalizing problems (Bagwell & Bukowski, 2018; Rubin, Bukowski, & Bowker, 2015). As children age, their social lives are characterized by increasingly spending time with peers (Collins & Laursen, 2004). Theories as the need-to-belong theory (Baumeister & Leary, 1995) and attachment theories point to the importance of close relationships as humans have the desire to form social connections, which are seen as necessary for survival. Close relationships are also fundamental to understand affect regulation (Mikulincer & Shaver, 2005). Unsatisfied relationships can create unpleasant feelings such as loneliness (Cacioppo et al., 2015) and positive close relationship can be a source of positive emotions (Ramsey & Gentzler, 2015).

Peers also play a role for individual’s self-esteem (Reitz, Motti-Stefanidi, & Asendorpf, 2016; Tetzner, Becker, & Maaz, 2017). For instance, a laboratory experiment demonstrated that being excluded from peers reduces self-esteem (Leary, Tambor, Terdal, & Downs, 1995). Previous longitudinal examinations suggest that associations between a social variable and self-esteem might be bidirectional, as peer acceptance can precede relative increases in self-esteem and vice versa (Reitz et al., 2016; Tetzner et al., 2017). Thus, self-esteem might be sensitive to the social environment (e.g., peer acceptance), and higher self-esteem, in turn, may facilitate social aspects of being liked. Self-esteem has been seen as a sociometer or internal gauge that responds to evaluations by others, and is sensitive to peer acceptance and rejection (sociometer theory; Leary & Baumeister, 2000). In this way, self-esteem tends to decrease when one experiences peer rejection or negative evaluations. Molloy, Ram, and Gest (2011) assessed academic and social self-concept twice a year for five years in third to fifth graders. Interestingly, variability in self-concept across the waves was related to several indicators of poorer social functioning including less engagement in social interactions and being less liked by peers. Despite the longitudinal and experimental studies on social functioning and self-esteem, to our knowledge, no study has examined if social or peer experiences are associated with *daily fluctuations* in self-esteem, which are closely tight to actual experiences.

***Externalizing problem behavior: Aggression towards others.*** Aggression refers to a set of behaviors that harms or hurts others. Maladaptive regulation of negative emotions is associated with aggressive behavior (Roberton, Daffern, & Bucks, 2012), not only in terms of problematic regulation of anger, but also sadness (Sullivan, Helms, Kliewer, & Goodman, 2010). Looking at daily experiences of affect, higher mean levels of NA as well as more variability in NA have been related to increased aggression and behavioral problems (e.g., truancy, destroying) in early adolescents (Neumann et al., 2011; Silk et al., 2003). Studies examining the daily experiences of PA do not unequivocally point to a clear relation between PA and aggression (Neumann et al., 2011), but Neumann et al. (2011) showed that aggressive behavior predicted prospective PA variability, rather than the opposite. Moreover, aggressive children and adolescents did not report different levels of happiness after imagined provocation by peers compared to a non-aggressive control group (Orobio de Castro, Merk, Koops, Veerman, & Bosch, 2005). Research has differentiated between various forms of aggression and shown validity of distinctions in aggression types by having different antecedents and consequences (Card, Stucky, Sawalani, & Little, 2008). Overt/direct aggression such as hitting and physical harm has been differentiated from relational/indirect aggression such as excluding others, gossiping, and spreading rumors (Card et al., 2008). Little is known about the specific types of aggression in relation to affective fluctuations.

Self-esteems relation with aggression is unclear: aggression has been associated with low self-esteem (Donnellan, Trzesniewski, Robins, Moffit, & Caspi, 2005), with high self-esteem (Hughes, Cavell, & Grossman, 1997), and has been unassociated with self-esteem (Kirkpatrick, Waugh, Valencia, & Webster, 2002). New perspectives have been developed. Researchers postulate that individuals with high self-esteem might be prone to showing aggression when their positive self-view is threatened by others; or postulate that especially individuals with a high though fluctuating self-esteem are prone to reveal aggressive behavior (Baumeister, Bushman, & Campbell, 2000). Indeed, Bukowski, Schwartzman, Santo, Bagwell, and Adams (2009) showed that children who have inflated positive views of their social competencies which are discrepant from other’s (objective) judgments, demonstrate more relational aggression and overt/physical aggression, with the strongest relation for relational aggression.

When considering actual fluctuations in self-esteem, within one level of self-esteem (i.e., high), the (in)stability of self-esteem can be a discriminating factor for aggressive behavior (Kernis, 2005). Lee (2014) showed that higher fluctuations in self-esteem, measured by the standard deviation of four repeated measures, was significantly correlated with higher levels of aggression in fifth and sixth graders. These findings require replication, especially since the study included only four repeated measures of self-esteem.

## Mean level as predictor of fluctuations

Whereas mean levels and fluctuations/variability can be treated as separate entities, their interrelatedness is informative. Both Van Roekel et al. (2016) and Neumann et al. (2011) found that higher mean levels of PA were associated with lower variability of PA in adolescents. For sadness, the reverse association was found (Neumann et al., 2011). Using an example from organizational psychology, higher mean PA was associated with less variation in PA across working days in managerial workers (Weiss, Nicholas, & Daus, 1999). With regards to self-esteem, a meta-analytic review by Okada (2010) concluded that the level of self-esteem is negatively related to self-esteem instability. However, the effect size of this association was weak. Moreover, researchers have suggested that mean levels should be included as a covariate in models of fluctuations, to rule out that associations with fluctuations are merely due to higher (or lower) mean levels (Russell, Moskowitz, Zuroff, Sookman, & Paris, 2007; Thompson et al., 2012). However, Neumann et al. (2011) highlighted that the intensity and variability of affect are not frequently examined together in models of symptomatology.

## Research aims

The aim of the present study was to assess how measures of internalizing problems, social functioning, and aggression were associated with mean scores and indices of variability of PA, NA, and self-esteem assessed within a sample of fifth- and sixth-grade boys and girls. Measures of PA, NA, and self-esteem were collected 20 times across four consecutive days. The correlates (i.e., internalizing problems, social functioning, and aggression) were assessed using a multi-informant method that relied on assessments from adolescents and their peers.

The following hypotheses were made: First, it was predicted that higher levels of internalizing problems would be associated with lower mean PA, higher mean NA, lower mean self-esteem, and more fluctuations in PA, NA, and self-esteem. Second, it was predicted that self-perceived and peer-perceived social functioning would be adaptive in terms of positive associations with mean PA and self-esteem and negative associations with mean NA and with fluctuations. Both self-perceived and peer-perceived social functioning were included, because both views do not necessarily lead to the same results (e.g., Reitz et al., 2016). Third, we assessed two different aspects of aggression (i.e., overt and relational) and predicted that both types of aggression would be associated with higher levels of NA. We had no clear prediction for the relation of aggression with PA. We expected that increased aggression would be associated with greater fluctuations in self-esteem, based on Lee (2014). Fourth, concerning the relation between mean and variability, we hypothesized that higher mean levels of PA and self-esteem would be associated with lower variability in PA, whereas the reverse association would be predicted for NA. As the present study is cross-sectional in nature, we make no predictions about the directionality of relationships. Although our approach considers affect as the outcome, we do not exclude associations in the opposite direction from affect to the other variables.

## Method

### Participants

Four participants were not included because there was no demographic information available and 14 participants were not included because they had missing values on major study variables. The included sample consists of 94 early adolescents (50 boys) in grades five (*N* = 45) and six (*N* = 49). The mean age of the participants was 10.8 years (*SD* = 0.7), ranging between 9 and 13 years (with 92 of the 94 adolescents aged between 10 and 12 years). The participants were from a single primary school in the Eastern Townships region of Québec in Canada. The school was attended by students from across the socioeconomic spectrum. English was the primary language used by children when interacting with their peers in these school contexts.

### Procedure

Potential participants were informed about the study at school and sent home with an invitation letter describing the study and giving parents the opportunity to give permission for participation in the study. The data collection for the adolescents consisted of two phases. In the first phase, participants completed questionnaires (e.g., internalizing symptoms, social functioning) and provided peer nominations in a collective session during school hours. In the second phase, an experience sampling method was applied. Herein, participants completed booklets including PA, NA, and self-esteem (and provided saliva samples) at fixed moments five times a day (30 minutes after waking, following arrival at school, late morning after morning recess, after lunch, and after afternoon recess) for four consecutive days (Tuesday to Friday). All assessments, except for the early morning assessments, took place at school. Research assistants were present to assist the adolescents. All questionnaires were completed in a paper-and-pencil format. As incentive, adolescents and parents received 20 Canadian dollars, an information session was provided, and a report on group findings was distributed. The study was approved by the Concordia University Human Research Ethics Committee (certification number UH2002-037-2).

### Measures

The present study is part of a broader project on the emotional and social functioning, peer relationships and stress levels of early adolescents. The measures included in the present study are described below.

**State positive and negative affect (Self-report).** To assess state PA and NA, participants completed a modified version of the state emotion scale by Diener and Emmons (1985) in repeated booklets. Instructions and items were adapted to the fifth and sixth grade reading level. The adapted version had four emotionally-valenced items to assess PA: *happy, pleased, enjoyment/fun, and joyful*. For NA, five items were used: *worried/anxious, frustrated, angry/hostile, unhappy*, and *depressed/blue*. Participants indicated how much they felt this way during the past 15 minutes on a 5-point scale ranging from *not at all* (1) to *extremely* (5).

**State self-esteem (Self-report).** To assess state self-esteem on the level of global self-worth, the following three items were administered in the repeated booklets: “I feel like I am a good person”, “I like myself”, “I feel happy with the way I am”. Participants had to indicate how they felt *right now*, using a 5-point scale ranging from 1 (*really disagree*) to 5 (*really agree*). These items were adapted from Harter’s (1982) Perceived Competence Scale for Children.

The internal consistency of PA, NA, and self-esteem measures was assessed with a multilevel technique developed by Nezlek (2017) to calculate an index of reliability when items on a scale are nested within occasions of measurement and occasions of measurement are nested within persons. This reliability observed for each scale was more than satisfactory. Specifically, the observed level of reliability was .93 for self-esteem; .88 for NA; .82 for PA.

Both mean and within-person variability of PA, NA, and self-esteem were examined. Within-person variability was defined as the within-person standard deviation of the variable across time (e.g., Okada, 2010), indicating the mean squared deviation from the adolescent’s mean level of the respective variable. Larger values stand for larger deviations from the person’s average level of affect. The standard deviation was calculated for each person for the repeated PA, NA, and self-esteem data. An average of 18.0 repeated assessments was provided (*SD* = 2.7) with a minimum of 9 and a maximum of 20. Number of completed experience sampling assessments was not significantly related to the baseline measure of internalizing problems, nor to the mean and standard deviation of PA, NA, and self-esteem, .002 < *r*’s < –.12, .25 < *p*’s < .98.

Moreover, for completeness, we also calculated another measure variability, i.e. the mean square successive difference (MSSD). This measure does not assess deviations from the mean, but changes from each assessment to the next assessment. As such, the difference between subsequent scores was calculated by subtracting consecutive scores from each other. Following the approach in Van Roekel et al. (2016), we only included differences during the day, not differences between evenings and mornings. Next, all difference scores were squared, and the mean of these squared differences scores was calculated. As the distribution of this measure was skewed, a square root transformation was used (MSSD_S). Again, larger values stand for more variability.

**Internalizing symptoms (Self-report and peer-report).** Internalizing symptoms were assessed using self-report and peer-report measures. *Self-report:* Participants were asked to indicate how well each of the items described them. The seven internalizing items addressed sadness, worry, annoyance, being nervous or tense, getting stressed a lot, loneliness, and hopelessness. Each item was rated on a 5-point scale ranging from 1 to 5 (*Never true, Rarely true, Sometimes true, Often true, Always true*). A well-defined minimal set of items that represent the construct were made to minimize the participants’ burden. Cronbach’s alpha for the internalizing scale was .81. *Peer-assessment*: An unlimited choice peer assessment procedure (see Bukowski, Cillessen, & Velasquez, 2012) was used to assess internalizing characteristics. As part of the larger study, each participant was given a list of all the participating children in their class and a list of several items that referred to different forms of social behavior, affective experience, or competence. Each participant was asked to indicate which of their participating classmates fit each characteristic or behavior in the list. The six items representing internalizing characteristics were the same as those used in the self-report measure of internalizing (except for the hopelessness item that was not assessed by peers). Each child was given a score on each item indicating how often they had been chosen for it by their same-gender classroom peers who were participating in the study. The regression-based procedures developed by Velásquez, Bukowski, and Saldarriaga (2013) were used to adjust the scores on each of the peer assessment items for biases that may result from variations in class size. The average of the six items adjusted for class size was used as the child’s score on this measure.

**Self-perceived social functioning (Self-report).** To assess self-perceived social functioning, the following four items were administered: “I have a lot of friends”, “I am always doing things with a lot of kids”, “I am popular with others my age”, “I am really easy to like”. These items are based on the social competence subscale of Harter’s (1982) Perceived Competence Scale for Children. They represent the basic domains of children’s interactions and relationships with peers. Using a 5-point scale ranging from 1 to 5 (*Never true, Rarely true, Sometimes true, Often true, Always true*) the participants rated each item according to whether it was a true description of their functioning with their peers. Cronbach’s alpha was .82.

**Peer-perceived social functioning (Peer-report).** As part of a larger study, participants completed three well-established questionnaires that were used to compute indices of competent functioning within the peer group (each is described in Bukowski et al., 2012). One was an unlimited choice positive nomination-based sociometric questionnaire. Each participant was shown a list of their participating same-gender classmates and was asked to indicate which of these peers they regarded as hir best friends. This questionnaire was used to compute a measure of acceptance and a measure of engagement in a reciprocated friendship. The number of times a child was chosen as a best friend was used as the nomination-based measure of the child’s acceptance among peers. A child was designated as having a best friend if any of the peers the child chose as a first- or second-, or third- best-friend had chosen the child as one of the first three friends. The second questionnaire was a rating scale sociometric measure. With this measure each student was given a list of their participating classmates and was asked to use a five-point scale to rate the extent to which they liked each one. On this scale a rating of 1 meant “do not like this person” and 5 meant “like this person very much”. The number of times a child received a rating of 5 was used as the rating scale-based measure of acceptance. The third questionnaire was a peer assessment measure in which each identified the participating same-gender peers who best fit each of two characteristics that are indices of popularity or status in the peer group. The items were “Someone who is liked by lots of people” and “Someone who is popular”. A score assigned to each participant on each characteristic based on the number of times the child was chosen for the characteristic by the other same-gender children. The procedures described by Velasquez et al. (2013) were used to correct the scores derived from these three questionnaires for potential biases that may result from variations in class size. A peer-perceived social functioning score was created by computing a factor score comprised on a weighted combination of the adjusted scores on the five measures taken from the three questionnaires, specifically the nomination-based acceptance score, the rating scale based measure of acceptance, the friendship reciprocity measure, and the two measures of peer-assessed popularity. The factor score was created with an exploratory principal components analysis that produced a single underlying latent factor. The weights assigned to the five measures were .77, .84, .75, .83 and .77, respectively.

**Aggression: Relational and overt aggression (Peer-report).** The peer-report procedure for aggression is identical to the peer-report procedure of internalizing problems. For relational aggression, the following three items were included: “Someone who tries to keep others out of the group when it’s time to play”, “Someone who talks bad about others behind their backs to hurt them”, and “Someone who when mad at someone, ignores or stops talking to him/her”. Three other items intended to measure overt aggression: “Someone who hurts others physically”, “Someone who hits pushes or shoves people”, and “Someone who gets involved in physical fights”.

## Results

### Preliminary analyses

Descriptive information and Pearson correlations between all study variables (i.e., general questionnaires and aggregated within-subject variables) were calculated using SPSS Statistics 24 and are reported in Tables 1 and 2, respectively. For PA, social functioning (both self-perceived and peer- perceived) revealed a significant, positive, association with mean PA levels. Internalizing symptoms, social functioning, and aggression were all not significantly related to PA variability (i.e., *SD* and *MSSD_S*). For negative affect, more self-reported internalizing symptoms and lower peer- perceived social functioning were associated with higher mean levels of NA. More self-reported internalizing symptoms as well as more overt aggression were associated with higher variability in NA (i.e., *SD* and *MSSD_S*). For self-esteem, lower levels of self-reported and peer-reported internalizing symptoms, and higher levels of self-perceived social functioning were correlated with higher mean self-esteem. Higher levels of self-reported and peer-reported internalizing symptoms, overt aggression, and lower levels of peer-perceived social functioning were correlated higher *SD* variability in self-esteem. For *MSSD_S* variability, higher levels of self-reported and peer-reported internalizing symptoms, and lower levels of peer-perceived social functioning were correlated higher self-esteem variability.

**Table 1:** Descriptive information of study variables.

	*M*	*SD*	*Min*	*Max*

PA mean (SR)	14.06	3.77	5.00	20.00
NA mean (SR)	6.99	2.70	5.00	17.50
Self-esteem mean (SR)	12.52	2.90	3.00	15.00
PA variability – SD	2.53	1.50	0.00	6.47
NA variability – SD	2.09	1.78	0.00	7.49
Self-esteem variability – SD	0.93	0.85	0.00	3.80
PA variability – MSSD_S	3.07	1.95	0.00	9.03
NA variability – MSSD_S	2.65	2.38	0.00	9.99
Self-esteem variability – MSSD_S	1.08	1.05	0.00	4.37
Internalizing symptoms (SR)	16.44	5.62	7.00	35.00
Internalizing symptoms (PR)	6.47	4.38	–0.40	20.20
Social functioning (SR)	13.55	3.87	4.00	20.00
Social functioning (PR)	–0.01	0.86	–1.54	2.18
Relational aggression (PR)	1.67	1.32	–0.86	6.86
Overt aggression (PR)	0.93	1.57	–0.47	8.20

*Note:* SR = self-report; PR = peer-report; variability – SD = within-person standard deviation of repeated assessments; MSSD_S = square root of mean square successive difference.

**Table 2:** Pearson correlations between all study variables.

	1.		2.		3.		4.		5.		6.		7.		8.		9.		10.		11.		12.		13.		14.	

1. PA mean (SR)	–																											
2. NA mean (SR)	–.09		–																									
3. Self-esteem mean (SR)	.53	***	–.23	*	–																							
4. PA SD	–.26	*	.24	*	–.15		–																					
5. NA SD	–.14		.65	***	–.25	*	.58	***	–																			
6. Self-esteem SD	–.25	*	.44	***	–.29	**	.28	**	.33	**	–																	
7. PA MSSD_S	–.24	*	.23	*	–.18		.91	***	.57	***	.25	*	–															
8. NA MSSD_S	–.18		.64	***	–.17		.62	***	.94	***	.31	**	.64	***	–													
9. Self-esteem MSSD_S	–.28	**	.40	***	–.28	**	.28	**	.27	*	.95	***	.27	*	.28	*	–											
10. Internalizing symptoms (SR)	–.07		.29	**	–.34	***	.14		.38	***	.23	*	.16		.35	***	.24	*	–									
11. Internalizing symptoms (PR)	–.15		.19		–.22	*	.10		.10		.34	***	.10		.11		.40	***	.32	**	–							
12. Social functioning (SR)	.34	***	–.07		.29	**	–.14		–.20		–.09		–.16		–.20		–.11		–.25	*	–.20		–					
13. Social functioning (PR)	.22	*	–.24	*	.18		–.17		–.17		–.37	***	–.14		–.15		–.38	***	–.23	*	–.37	***	.48	***	–			
14. Relational aggression (PR)	.08		.07		.06		.11		.08		–.07		.15		.07		–.02		.11		.16		.14		.17		–	
15. Overt aggression (PR)	–.05		.19		.06		.19		.23	*	.21	*	.13		.28	*	.19		.06		.09		–.03		–.20	*	.20	*

*Note:* SR = self-report; PR = peer-report; SD = within-person standard deviation of repeated assessments; MSSD_S = square root of mean square successive difference. * *p* < .05; ** *p* < .01; *** *p* < .001

### Regression Analyses

Regression analyses were conducted in Mplus version 7 using maximum-likelihood estimation with robust standard errors. Seven regression analyses were conducted to examine associations with mean PA, NA, and self-esteem; and with variability in NA and self-esteem (i.e., *SD* and *MSSD_S*) after controlling for other correlates. Besides gender, variables that revealed a significant correlation with the dependent variable were included as predictor (i.e., self-reported internalizing problems, peer-reported internalizing problems, self-perceived social functioning, peer-perceived social functioning, relational aggression, and overt aggression). For the analyses examining relations with within-person variability, the mean level of the respective variable was also included to examine associations with variability above and beyond the contribution of mean levels.

***Positive affect: mean (Table 3)*.** Higher levels of self-perceived social functioning were the only significant predictor of mean PA. Although the peer measure of social functioning was also positively related to mean PA in the zero-order correlations, this association disappeared after controlling for the other variables. No regression analysis was conducted for within-person variability in PA as there were no significant correlations with the predictors.

**Table 3:** Regression analysis for positive affect (mean).

	Positive Affect Mean			

	*β*	*Standard error*	*p*	*R^2^*

Gender	–0.05	0.19	.81	
Social functioning (SR)	**0.30**	0.10	.004	
Social functioning (PR)	0.08	0.11	.49	.12

*Note: N* = 94. SR = self-report; PR = peer-report. Gender: boys coded as 0 and girls coded as 1. Regression coefficients indicate the results for predictors and criterion variable standardized (except for gender). Significant regression coefficients are in bold.

***Negative affect: mean and fluctuations (Table 4)*.** As predicted, higher levels of self-reported internalizing problems were significantly related to higher mean NA. Moreover, higher peer-perceived social functioning was significantly associated with lower mean NA. For *SD* variability, higher levels of internalizing problems did significantly relate to more variability in NA. However, overt aggression did not remain significant in the present regression analysis, taking into account the contribution of the other variables. As predicted, the higher the mean levels of NA, the more NA fluctuated. For *MSSD_S* variability, only mean levels of NA did significantly relate to more variability.

**Table 4:** Regression analyses for negative affect (mean and within-person variability).

	Negative Affect Mean					Negative Affect SD					Negative Affect MSSD_S			

	*β*	*Standard error*	*p*	*R^2^*		*β*	*Standard error*	*p*	*R^2^*		*β*	*Standard error*	*p*	*R^2^*

Gender	0.06	0.21	.78		Gender	0.14	0.16	.37		Gender	0.10	0.17	.54	
Intern. symp. (SR)	**0.24**	0.09	.005		Intern. symp. (SR)	**0.19**	0.09	.04		Intern. symp. (SR)	0.16	0.10	.12	
Social funct. (PR)	–**0.19**	0.10	.047	.12	Overt aggr. (PR)	0.12	0.07	.09		Overt aggr. (PR)	0.16	0.08	.06	
					NA MEAN	**0.56**	0.10	<.001	.47	NA MEAN	**0.56**	0.10	<.001	.46

*Note: N* = 94 for mean and SD; *N* =85 for MSSD_S. SR = self-report; PR = peer-report; SD = standard deviation; MSSD_S = square root of mean square successive difference. Gender: boys coded as 0 and girls coded as 1. Regression coefficients indicate the results for predictors and criterion variable standardized (except for gender). Significant regression coefficients are in bold.

***Self-esteem: mean and fluctuations* (*Table 5*).** As predicted, higher levels of internalizing symptoms were associated with lower mean self-esteem. This only held for self-rated internalizing symptoms in the regression analysis. For *SD* variability in self-esteem, lower levels of peer-perceived social functioning were significantly associated with more self-esteem variability. However, internalizing symptoms and aggression were no longer significant in the regression analysis. Finally, lower mean levels of self-esteem were associated with more fluctuations. For *MSSD_S* variability in self-esteem, lower levels of peer-perceived social functioning as well as higher levels of peer-reported internalizing symptoms were significantly associated with more variability. Lower mean levels of self-esteem were not significantly associated with more variability.

**Table 5:** Regression analyses for self-esteem (mean and within-person variability).

	Self-Esteem Mean					Self-Esteem SD					Self-Esteem MSSD_S			

	*β*	*Standard error*	*p*	*R^2^*		*β*	*Standard error*	*p*	*R^2^*		*β*	*Standard error*	*p*	*R^2^*

Gender	–0.07	0.20	.73		Gender	–0.15	0.21	.49		Gender	–0.22	0.22	.32	
Intern. symp. (SR)	**–0.25**	0.11	.02		Intern. symp. (SR)	0.05	0.10	.62		Intern. symp. (SR)	0.06	0.11	.60	
Intern. symp. (PR)	–0.08	0.11	.44		Intern. symp. (PR)	0.22	0.12	.07		Intern. symp. (PR)	**0.28**	0.12	.03	
Social funct. (SR)	0.21	0.13	.10	.17	Social funct. (PR)	**–0.21**	0.09	.02		Social funct. (PR)	**–0.22**	0.10	.04	
					Overt aggr. (PR)	0.14	0.10	.16		Self-est. MEAN	–0.17	0.10	.10	.25
					Self-est. MEAN	**–0.20**	0.10	.04	.25					

*Note: N* = 94 for mean and SD; *N* = 87 for MSSD_S. SR = self-report; PR = peer-report; SD = standard deviation; MSSD_S = square root of mean square successive difference. Gender: boys coded as 0 and girls coded as 1. Regression coefficients indicate the results for predictors and criterion variable standardized (except for gender). Significant regression coefficients are in bold.

## Discussion

In a sample of early adolescents in grade five and six, state PA, NA, and self-esteem were assessed five times a day for four consecutive days. The means and within-person variability (i.e., *standard deviation* and *the mean square successive difference*) in PA, NA, and self-esteem were examined in relation to internalizing symptoms, social functioning, and two types of aggression. In the discussion, we will focus on the results from the regression analyses in which associations are controlled for the contribution of other variables.

### Internalizing symptoms

Internalizing symptoms were not significantly related to both mean and within-person variability in PA. This might appear counterintuitive given that reduced pleasurable experiences are a core aspect of depression. This result also contradicts research in which PA variability was found to be positively associated with depressive symptoms (Gruber et al., 2013). Speculatively, if the measure of internalizing symptoms had a broader content including anhedonia, a relationship might appear. In line with this, recent findings revealed that anhedonia is related to lower daily PA means and higher PA variance in late adolescence/emerging adulthood (Heininga, Van Roekel, Ahles, Oldehinkel, & Mezulis, 2017). Moreover, internalizing problems include both depressive and anxiety symptoms. Clark and Watson’s (1991) tripartite model of anxiety and depression states that low PA is particularly characteristic of depression. Nevertheless, some authors have indicated or found that several anxiety problems are also characterized by low PA, though to a smaller extent than depression (Gilbert, 2012; Watson & Naragon-Gainey, 2010; Watson & Stasik, 2014).

Participants who reported more internalizing problems did experience higher mean NA as well as more fluctuations in NA (i.e., within-person standard deviation). Although it was unexpected that internalizing symptoms were only related to NA, not PA, variability, it is partially in line with a meta-analysis showing that dynamics in negative emotions are more strongly related to general psychological well-being than dynamics in positive emotions (Houben et al., 2015). The finding for mean NA is in line with increased negative feelings as a core aspect of depression and could be expected to be even stronger at an older age given that Larson et al. (1990) found larger affective differences between dysphorics and non- dysphorics in early secondary compared to late elementary school adolescents. Moreover, the present result affirms the scarce evidence from research on fluctuations in NA that included subjects from elementary school (Larson et al., 1990; Neumann et al., 2011). The result does not suggest that flat mood is desirable. Rather, emotion dysregulation implies that too much fluctuation is not adaptive (as is high average NA).

Concerning self-esteem, self-reported internalizing symptoms were related to lower mean self-esteem, which is in line with the widespread evidence that low self-esteem predicts depression (e.g., Sowislo & Orth, 2013). Moreover, peer-reported internalizing problems predicted more fluctuations in self-esteem (i.e., greater *MSSD_S* of self-esteem). While we cannot make any conclusions about the direction of these relations, previous longitudinal research in adults indicate that the amount of self-esteem fluctuations can precede a relative increase in depressive symptoms (Franck & De Raedt, 2007; Franck et al., 2016), and thus play a role in the etiology of depressive symptoms. Surprisingly, in the regression analysis, internalizing symptoms were only significantly related to self-esteem variability in terms of *MSSD*, not the within-person standard deviation (i.e., *SD*). Nevertheless, the standardized coefficient was 0.22, albeit non-significant (*p* = .07).

### Self-perceived and peer-perceived social functioning

Continuing the previous paragraph on self-esteem, most importantly, the present study extends the literature by revealing that peers’ perceptions about popularity or liking were relevant for the fluctuations in adolescents’ self-esteem. Speculatively, this may be because the judgments of peers are a reflection of daily threats of the self-view in terms of daily social feedback or ways of interaction and cause unstable feelings of self-esteem. In turn, being unstable in self-esteem might elicit interpersonal styles that are not liked by peers. Our finding is in line with previous research in the end of elementary school examining variability in self-esteem over long intervals (vs. across days), which showed that more variability in levels of social self-concept were associated with poorer social functioning (Molloy et al., 2011). Whereas research on self-esteem instability has often been conducted in students or adults, the present study extends these findings to early adolescents, and importantly, it shows the relevance of fluctuations above and beyond the contribution of mean levels of self-esteem. This suggests that at different levels of self-esteem, early adolescents differ in how sensitive or responsive they are to daily experiences that challenge their positive self-views (see also, for instance, in Franck et al., 2016).

Considering affect, self-perceived and peer-perceived social functioning were of relevance for mean PA and NA, respectively. Also, social functioning was the only significant predictor for mean PA. This result is in line with research showing that close relationships play a key role in positive affective experiences (Ramsey & Gentzler, 2015). One of the explanations is that close relationships contribute to interpersonal affect regulation in which people regulate the other person’s affect or in which each other’s emotions are coregulated or linked (Butler & Randall, 2012). Researchers have stressed the bidirectionality of the relationship-affect relationship (Ramsey & Gentzler, 2015) as positive affect may also facilitate positive social interactions. As social relationship with peers gain importance throughout adolescence, perceiving oneself as a likable person may even become more important for adolescents’ pleasurable and dejected feelings later in adolescence, and accordingly increase the risk for adolescents who are unsure about their social capacities or who are not integrated in peer groups.

### Aggression

Aggression was not significantly related to mean nor variability in PA nor NA in our regression analyses. This was especially unexpected for NA. As we examined the unique association of aggression with variability in NA, the results show that aggression does not significantly contribute to NA after controlling for internalizing problems. Moreover, the peer-reported nature of aggression may underestimate aggression outside school. Mean and variability in NA have been associated with adolescents’ externalizing problem behavior such as stealing and destroying (Silk et al., 2003). However, longitudinal results in early adolescents showed that across different analyses and different types of emotions, anger is the most relevant for aggression (Neumann et al., 2011) and thus more refinement in negative affect may be required.

Concerning self-esteem, aggression was unrelated to mean levels of self-esteem, as predicted. This is a replication of the zero-order correlations reported by Lee (2014) in the same grades but with a smaller number of repeated measures of self-esteem. Unexpectedly, aggression was also not significantly related to fluctuations in self-esteem in the regression analysis. However, the significant zero-order correlation is in line with Lee (2014) who found a positive correlation between aggression and fluctuations in self-esteem.

### Association between mean levels and within-person variability

As predicted, the present findings showed that high mean NA is likely to be combined with more fluctuations in NA, whereas high PA is likely to be combined with less fluctuations in PA. The association between mean levels and within-person variability was clearly stronger for NA than PA. These results form an independent replication of the associations reported in Neumann et al. (2011) and Van Roekel et al. (2016) and suggest that interventions aimed at promoting PA and reducing NA might have the (beneficial) side effect of leading to changes in variations, or vice versa. Experimental examinations are needed to derive causal conclusion.

### Strengths, limitations, and future directions

A major strength of the present study is the use of the event-sampling procedure which requires minimal retrospective recall as assessments are closer to actual experiences than one-time general questionnaire reports or end-of-day reports. Next, the booklets were completed at roughly the same moment each day and at the same moment for all participants, controlling for diurnal variations in affect. Moreover, two indicators of within-person variability were examined. Finally, in all regression analyses examining fluctuations, mean levels were controlled for. Therefore, the found associations with fluctuations cannot be explained by shared variance with mean levels.

A first limitation is that there were no repeated assessments during late afternoons, evenings, and weekends. This might confound the results given that adolescents’ PA varies according to the day of the week, showing a peak on Saturday (Csikszentmihalyi & Hunter, 2003). Follow-up analyses in our sample revealed that PA (and self-esteem) did not significantly differ across the four weekdays. However, NA was significantly higher on Tuesday compared to the three following days. Secondly, we examined PA and NA without taking into account the cognitive and behavioral strategies adolescents use to regulate their affect. Research indicates that emotions as well as the strategies that are used to regulate them predict psychopathology (Aldao, Nolen-Hoeksema, & Schweizer, 2010). Increased fluctuations in daily affect can be a reflection of the use of inadequate affect regulation strategies such as dampening thoughts in response to PA and rumination in response to NA. Thirdly, we did not differentiate between the different types of emotions (e.g., sadness, anger; happy, pleased). For aggression, especially anger might be of relevance (Neumann et al., 2011; Orobio de Castro et al., 2005). However, a differentiation in emotions could be less important for the analyses with internalizing symptoms as different negative emotions have been non-specifically related to depressive and anxiety symptoms in an adolescent sample (Neumann et al., 2011).

It is important to note that participants were adolescents at the end of primary school, who tend to experience fewer depressive symptoms than older adolescents. It is plausible that results are different later in adolescence, such that replication in older age groups is needed. Furthermore, the found associations do not necessarily generalize to other developmental phases as previous research indicated that specific associations with self-esteem can change across the lifespan (Tetzner et al., 2017). In terms of the direction of relations, we cannot conclude whether variables are either antecedent to or a consequence of each other. In order to do so, longitudinal research in which the daily diary assessments and general questionnaires are carried out at more distinct moments is required.

## Conclusion

The present paper addressed several shortcomings in the literature. The distinction between mean and variability in affect has not been extensively examined in early adolescence. The study did not solely focus on either PA or NA but examined both. To our knowledge, thus far no studies have examined whether social experiences are associated with within-person *variability* in self-esteem. Multiple types of aggression were acknowledged, and the study formed an independent replication of the singly previous study on different types of aggression in relation to variability in self-esteem.

First, internalizing symptoms did not relate to a general pattern of increased affective variability, but only to variability in NA, not PA. Second, social functioning was related to mean PA and NA, confirming the importance of peer relationships for adolescents’ daily affective functioning. Third, importantly, the more positive the peers, not adolescents themselves, evaluated the adolescent’s social functioning, the less prone adolescents were to fluctuations in self-esteem. Fourth, how peers evaluated the adolescent’s internalizing symptoms was significantly associated with self-esteem variability in terms of moment-to-moment variations. Fifth, unexpectedly, aggression was not significantly associated with daily experience of PA, NA, nor self-esteem in all regression analyses. Finally, mean and variation were not orthogonal, such that higher mean NA related to more NA fluctuations, whereas higher mean PA and self-esteem related to less fluctuations.
